# Effect of Co-Inoculation of *Candida zemplinina*, *Saccharomyces cerevisiae* and *Lactobacillus plantarum* for the Industrial Production of Negroamaro Wine in Apulia (Southern Italy)

**DOI:** 10.3390/microorganisms8050726

**Published:** 2020-05-13

**Authors:** Maria Tufariello, Vittorio Capozzi, Giuseppe Spano, Giovanni Cantele, Pasquale Venerito, Giovanni Mita, Francesco Grieco

**Affiliations:** 1 Institute of Sciences of Food Production, National Research Council, 73100 Lecce, Italy; giovanni.mita@ispa.cnr.it; 2 Institute of Sciences of Food Production, National Research Council, c/o CS-DAT, Via Michele Protano, 71121 Foggia, Italy; vittorio.capozzi@ispa.cnr.it; 3 Department of the Sciences of Agriculture, Food and Environment, University of Foggia, 71121 Foggia, Italy; giuseppe.spano@unifg.it; 4 Azienda Vinicola Cantele. S.P. 365 Km 1, 73010 Guagnano, Italy; giannicantele@cantele.it; 5 Center for Research, Experimentation and Training in Agriculture “Basile Caramia”, 70010 Locorotondo, Italy; pasqualevenerito@crsfa.it

**Keywords:** wine, mixed starter cultures, multi-strains, *Lactobacillus plantarum*, *Saccharomyces cerevisiae*, *Candida zemplinina*, *Starmerella bacillaris*, autochthonous yeast, alcoholic fermentation, malolactic fermentation, commercial-scale

## Abstract

The employment of multi-species starter cultures has growing importance in modern winemaking for improving the complexity and wine attributes. The assessment of compatibility for selected species/strains at the industrial-scale is crucial to assure the quality and the safety associated with fermentations. An aspect particularly relevant when the species belong to non-*Saccharomyces*, *Saccharomyces* spp. and malolactic bacteria, three categories with different biological characteristics and oenological significance. To the best of our knowledge, the present report is the first study regarding the utilization of a combined starter culture composed of three strains of non-*Saccharomyces*, *Saccharomyces cerevisiae* and *Lactobacillus plantarum* for production of wine at the industrial scale. More in-depth, this work investigated the oenological potential of the autochthonous characterized strains from the Apulian region (Southern Italy), *Candida zemplinina* (syn. *Starmerella bacillaris*) 35NC1, *S. cerevisiae* (NP103), and *L. plantarum* (LP44), in co-inoculation following a complete scale-up scheme. Microbial dynamics, fermentative profiles and production of volatile secondary compounds were assessed in lab-scale micro-vinification tests and then the performances of the mixed starter formulation were further evaluated by pilot-scale wine production. The above results were finally validated by performing an industrial-scale vinification on 100HL of Negroamaro cultivar grape must. The multi-starter formulation was able to rule the different stages of the fermentation processes effectively, and the different microbial combinations enhanced the organoleptic wine features to different extents. The findings indicated that the simultaneous inoculation of the three species affect the quality and quantity of several volatile compounds, confirming that the complexity of the wine can reflect the complexity of the starter cultures. Moreover, the results underlined that the same mixed culture could differently influence wine quality when tested at the lab-, pilot- and industrial-scale. Finally, we highlighted the significance of employment non-*Saccharomyces* and *L. plantarum*, together with *S. cerevisiae*, autochthonous strains in the design of custom-made starter culture formulation for typical regional wine production with pronounced unique quality.

## 1. Introduction

The spontaneous conversion of grape must into wine is a composite process of microbial origin denoted by the consecutive growth of different species of oenological yeasts and bacteria, associated with the grapes and the cellar plants [1,2]. The first stage of the alcoholic fermentation (AF) is performed by several species of non-*Saccharomyces* yeasts (such as *Candida spp., Hanseniaspora spp.,* and *Pichia spp*.) and then strains belonging to *Saccharomyces cerevisiae* species complete the fermentation process [3].

The malolactic fermentation (MLF) is a secondary fermentation process that naturally occurs at the end of the AF, it being generally promoted by lactic acid bacteria (LAB) denoted by high tolerance to ethanol and pH tolerant LAB. Even though, the *Oenococcus oeni* is presently the broadest species utilized in wine production [4], strains belonging to the *Lactobacillus plantarum* species have demonstrated that they can also be suitable as MLF starter cultures [5,6]. The inoculation with selected LAB starter cultures could warrant a successful MLF and avoid risks for the consumer’s health, such as the production of ethyl carbamate and biogenic amines by wild LAB strains [7,8,9,10].

The effect of the non*-Saccharomyces* species during the AF is significant since they play a pivotal role in defining the composition of wine aroma [11]. Recent investigations have demonstrated that these yeasts can be used to modify and enhance the aromatic complexity of wines [12,13]. Several non*-Saccharomyces* yeasts are commercialized as oenological starter cultures (e.g., *Metschnikowia pulcherrima*, *Torulaspora delbrueckii*, *Lachancea thermotolerans, Candida zemplinina*, and *Pichia kluyveri)*, suggested for the use in mixture with *S. cerevisiae* strains [14,15].

Among non*-Saccharomyces* yeasts, *C. zemplinina* (syn. *Starmerella bacillaris*) is denoted by a strong fructophilic nature and by a low production of ethanol [14,16,17]. The enological significance of *C. zemplinina* strains used in combination with *S. cerevisiae* has been demonstrated [18,19,20,21], it being wines produced by the above-mixed starter characterized by higher amounts of glycerol and esters [17,22]. Moreover, the selective fructose consumption, after initial growth of *C. zemplinina* strains alleviated the osmotic stress suffered by *S. cerevisiae* cells [19]. During a recent investigation, we have investigated the technological and fermentative features of sixteen distinct *C. zemplinina* strains isolated from spontaneous fermentation in Apulia [23]. The analysis of produced wines showed that all the strains had fructophilic character and produced low amounts of acetic acid and hydrogen sulphide. The examination of produced volatile compounds indicated that *C. zemplinina* 35NC1 strain was able to enhance the aromatic complexity of wine, thus suggesting this biotype as a candidate for the preparation of a mixed starter culture together with a strain of *S. cerevisiae*.

Increasing interest has been direct to the understanding of the interactions between *S. cerevisiae* [24,25,26] and non-*Saccharomyces* [27,28,29,30] strains with different lactic acid bacteria (LAB), such as *L. plantarum* [5,31]. However, none of the above studies has tested the relation among the above three microbes by carrying out the vinification at the industrial scale. The lack of knowledge about the species-specific impact through the industrial winemaking process represents a limitation for the technological transfer.

In the present investigation, we assessed the oenological performance and the compatibility of mixed starter formulation composed of three Apulian autochthonous starter strains belonging to the *S. cerevisiae* [32]*, C. zemplinina* [23] and *L. plantarum* [33] strains by performing lab- and pilot-scale vinification tests. The “mixed” formulation was further validated during the vintage 2017 ad 2018 by carrying out two different large-scale vinifications of Negroamaro grape must. To the best of our knowledge, this study described, for the first time, the fermentative performance of a non-*Saccharomyces*/*Saccharomyces*/malolactic bacteria mixed starter formulation for the industrial production of a red wine.

## 2. Materials and Methods 

### 2.1. Microbial Strains

*Saccharomyces cerevisiae* NP103 and *Candida zemplinina* 35NC1 strains have been previously described after their isolation from spontaneous fermentation of Negroamaro grapes [23,32] and they are deposited in Agro-Food Microbial Culture Collection of ISPA (http://www.ispacnr.it/collezioni-microbiche). The yeast strains were sub-cultured on YEPD (10 g/L yeast extract, 20 g/L peptone, 20 g/L glucose, 20 g/L agar) and maintained at −80 °C in glycerol 50% [34]. *Lactobacillus plantarum* strain LP44 was isolated from spontaneous vinification in Apulia and, after its characterization [33], it was deposited in the collection of the Department of the Sciences of Agriculture, Food and Environment (University of Foggia). The LAB strain was cultured in MRS broth and maintained at −80 °C in glycerol 50%.

### 2.2. Microbial Population Analyses

The microbial dynamics during lab-scale fermentations were assessed by carrying out specific agar plate assay. Decimal dilutions of each sample were applied on WL Nutrient Agar (WLN medium; Oxoid Limited, Basingstoke, UK) and Lysine Agar (LA medium; Oxoid, UK) both added with 0.1 g/L ampicillin, to enumerate the *S. cerevisiae* or the *C. zemplinina* populations, respectively [25]. The *L. plantarum* populations were counted by spreading sequential dilutions of must sample on MRS agar pH 4.8 (Merck, Darmstadt, Germany) supplemented with 2% tomato juice and 0.05 g/L nystatin. Genomic DNA was extracted according to Tristezza et al. [35]. The identification of yeast populations was carried out by performing specific molecular assays; respectively, the ITS pattern examination for *C. zemplinina* [12] and the analysis of inter-delta profiles of *S. cerevisiae* were analyzed as described by Tristezza et al. [36].

### 2.3. Lab-Scale Tests

The micro-fermentation assays were carried out in sterilized must from Negroamaro grape (sugars 220 g/L, pH 3.4, assimilable nitrogen concentration 110.11 g/L). The must was firstly centrifuged (10 min at 8000× g) and then sterilized by membrane filtration (0.45 mm Ø membrane). Four hundred millilitres of treated must were aliquoted in sterile Erlenmeyer flasks added with malic acid in order to obtain a final concentration of 2.5 g/L and then we evaluated the ability of *C. zemplinina* isolate to drive the fermentation process in combination with *S. cerevisiae* and *L. plantarum.* Each flask was inoculated with the following concentration of yeast pre-cultured in the same must for 48 h at 25 °C [34,37]. The fermentation trials were carried out in triplicate. In Negroamaro must, *S. cerevisiae* NP103 and *C. zemplinina* 35NC1 were added at 10^4^ CFU/mL and 10^6^ CFU/mL respectively, to obtain a ratio of 1:100 while *S. cerevisiae* NC103 and *L. plantarum* were inoculated at 10^4^ CFU/mL and 10^8^ CFU/mL, respectively. Fermentation kinetics were daily recorded by gravimetric analysis and, when the weight was constant, the samples were collected and stored at −20 °C.

### 2.4. Pilot-Scale Vinification

The selected strains were tested in pilot-scale fermentation trials. The vinification was carried out in duplicate in an experimental cellar using sterile stainless steel 100-L vessels [37]. Ninety litres of Negroamaro must (240 g/L of total sugars, pH 3.52, 232 mg/L of assimilable yeast nitrogen, added with 20 g/hL of potassium metabisulphite) were inoculated with the *S. cerevisiae* NC103, *C. zemplinina* 35NC1, and *L. plantarum* LP44 using the same ratio adopted to perform the lab-scale vinification. The kinetics of the alcoholic fermentation process was monitored daily by measuring the density of the fermenting must. Samples of must and wines were collected as single replicates and stored at −20 °C for further analyses.

### 2.5. Industrial-Scale Vinification

Industrial-scale vinifications were carried out in a 150,000 L stainless steel vessel. To promote must fermentation, the preliminary inocula were produced, moved to the winery and then employed as starters [36]. The mixed starters cultures of *C. zemplinina* 35NC1, *S. cerevisiae* NP103, and L. *plantarum* LP44 respectively corresponding to 7 × 10^11^ CFU/hL, 7 × 10^9^ CFU/hL and 7 × 10^14^ CFU/hL were mixed with 300 kg of grape must for 6 hours. Then, the microbes-must mix was added to 10 tons of Negroamaro must, added with 20 g/hL of potassium metabisulphite, during the vintages 2017 (222.8 g/L of total sugars, pH 3.35, yeast assimilable nitrogen 139.8 g/L) and 2018 (226.1 g/L of total sugars, pH 3.29, yeast assimilable nitrogen 123.1 g/L). The alcoholic fermentation processes were both performed at 25 °C, and they were daily monitored by determining the must density and the concentration of reducing sugars. Must and wine samples were taken as single replicates for the microbiological and chemical analysis.

### 2.6. Wines Chemical Analyses

The wine chemical profile defined by some important parameters such as ethanol, pH, sugars, volatile and total acidity, malic, lactic, and tartaric acids and others was evaluated by Fourier transform infrared spectroscopy (FTIR) by employing the Winescan Flex (FOSS Analytics, Hilleroed, DK). After centrifugation at 8000 rpm for 10 min, the samples were analyzed according to the supplier’s instructions. Free volatile compounds were extracted by means of solid-phase extraction (SPE) [38]. Fifty millilitres of the wine were added with 2-octanol, as internal standard, at a final concentration of 100 mg/L and loaded onto Strata X resins pre-packed in 500 mg cartridges (Phenomenex, Torrance, CA, USA) previously activated by rinsing with 8 mL dichloromethane, 8-mL methanol, and 8-mL water, at around 2 mL/min. The cartridge was then washed with water, followed by 5-mL dichloromethane for recovering free aroma compounds. The dichloromethane fractions were dried over anhydrous Na_2_SO_4_ and concentrated under a stream of pure N_2_ to the volume of 0.5 mL before gas chromatography-mass spectrometry (GC-MS) analysis [32]. Sample (1 µL) was injected into a DB-WAX capillary column (60 m × 0.25 mm I.D., 0.25 mm film thickness) and then analyzed with a 6890N series gas chromatograph equipped with an Agilent 5973 mass spectrometer selective detector (Agilent, Santa Clara, CA, USA). A split/splitless injector was used in the splitless mode, the injector temperature was 250 °C, and the injected volume was 2 mL. The column oven temperature was initially held at 40 °C, then it was programmed to 200 °C at 4 °C/min, with a final holding time of 20 min. Spectra were recorded in the electron impact mode (ionization energy, 70 eV) in a range of 30–500 amu at 3.2 scans/s. The identification of the volatile compounds was achieved by comparing mass spectra with those of the data system library (NIST 98, P > 90%), with the retention data of commercially available standards and MS data reported in the literature. Quantification analysis was based on the principle that the component peak area is proportional to the amount of the analyte present in the sample. The quantification was carried out following the internal standard quantification method. The kinetics of the fermentations were monitored daily by gravimetric determinations, recording the weight decrease caused by the release of CO_2_. 

The qualitative hydrogen sulphide production was evaluated by the blackening of the PbAcO paper inserted between the plug and inner wall of the Erlenmeyer, above the level of the liquid. Based on the results obtained, the isolates were classified as high (+++), medium (++), low (+), and no (-) sulphide producers [12].

### 2.7. Statistical Analysis

One way analysis of variance (ANOVA) and principal component analysis were carried out using the STATISTICA7.0 software (StatSoft software package, Tulsa, OK, USA).

## 3. Results and Discussion

The aim of the present investigation was the study of the enological performances of a mixed starter, consisting of three autochthonous strains, i.e., *C. zemplinina* strain 35NC1, *S. cerevisiae* strain NP103 and *L. plantarum* strain LP44. These three strains, all selected from Apulian spontaneous fermentations, were previously genetically characterized and their fermentative performances were studied singularly at lab-scale. *C. zemplinina* strain 35NC1 was able to enhance the volatile profile of produced wine demonstrating (i) a low producer of acetic acid and hydrogen sulphide, (ii) inability to decarboxylate several amino acids, (iii) fructophilic character and significant glycerol production [23]. The *S. cerevisiae* strain NP103 was proposed as a fermentation starter because of its properties: low production of acetic acid, no synthesis of hydrogen sulphide, total sugar conversion during fermentation, significant production of volatile molecules responsible for wine aroma [32]. The technological characterization of *L. plantarum* LP44 indicated that this strain possesses: (i) tolerance to low pH, high sugar and high ethanol content, (ii) ability to survive after lyophilization stress and (iii) capacity to grow/survive and carry out MLF in grape must [33].

At the best of our knowledge, we first validated the use of the above a multi-species starter cultures non-*Saccharomyces*/*Saccharomyces*/malolactic bacteria for the industrial wine production. Furthermore, we provided original findings allowing improved exploitation of microbial diversity in the design of tailored starter culture for the production of typical wines with pronounced unique quality.

### 3.1. Lab-Scale Trials

**Figure 1:**
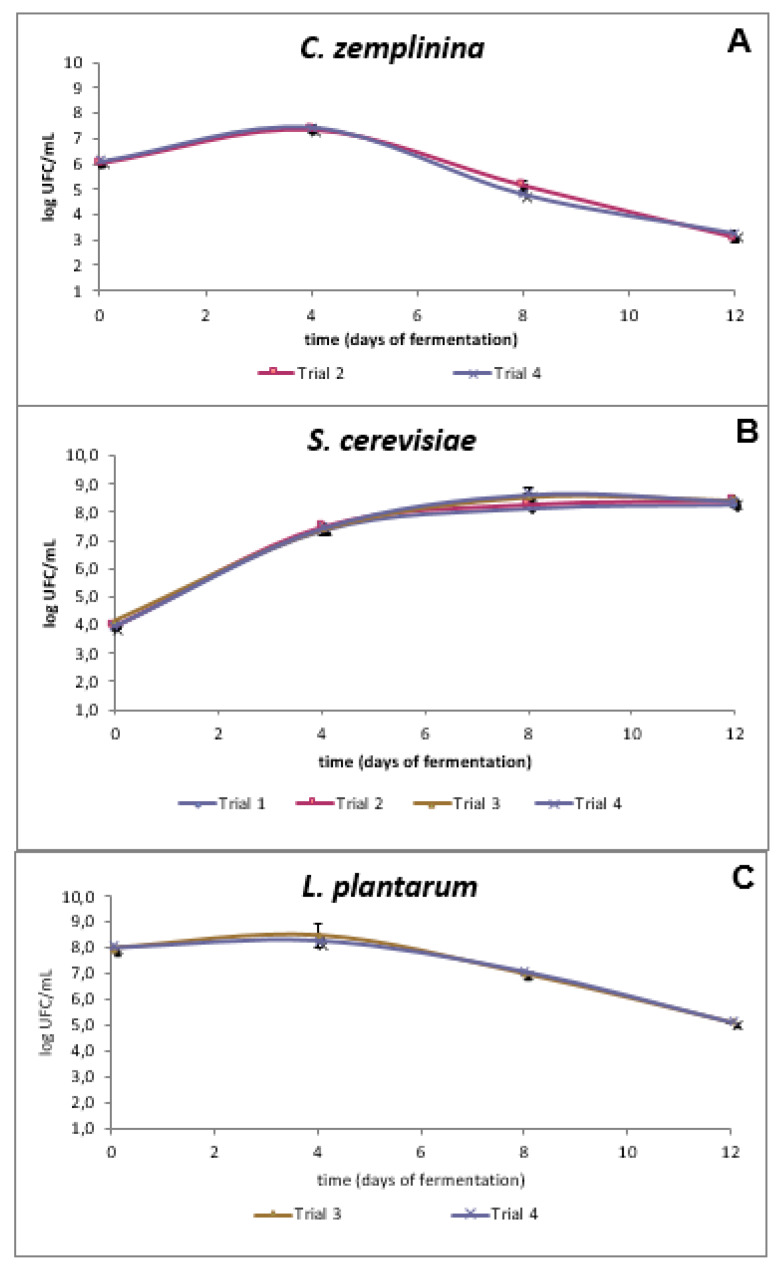
Viable cell count of *C. zemplinina* 35NC1 strain (**A**), *S. cerevisiae* NP103 strain (**B**), and *L. plantarum* LP44 strain (**C**) populations isolated throughout the lab-scale vinification tests.

As first step, four different vinifications of Negroamaro sterilized must were set up at the lab-scale. The Trial 1 was inoculated as control with the *S. cerevisiae* NP103 strain alone. The second vinification, denoted as Trial 2, was added with the NP103 strain together with *C. zemplinina* 35NC1 [23]. In Trial 3 the *S. cerevisiae* strain was co-inoculated with *L. plantarum* LP44 strain. Finally, the fourth vinification (Trial 4) was inoculated by the joint addition of all the three above mentioned starter strains, simultaneously. All the vinifications completed the alcoholic fermentation process in 12 days. After 72 h of fermentation (Figure 1A), *C. zemplinina* 35NC1 showed cell concentration increase in both Trial 2 and 4 (7.35 × 10^6^ and 7.47 × 10^6^ CFU/mL, respectively). Then, the above yeast concentration decreased, until the end of the fermentation, to 3.09 × 10^6^ CFU/mL (Trial 2) and 3.28 × 10^6^ CFU/mL (Trial 4). *S. cerevisiae* NP103 reached in all the four lab-scale vinifications its maximum concentration (8 × 10^6^ CFU/mL) after six days post-inoculation and then remained constant until the end of the fermentation (Figure 1B). The strain of *L. plantarum* LP44 showed a similar trend in both Trial 3 and 4 (Figure 1C). In this case, the bacterial starter presented a maximum concentration of 8.48 × 10^8^ CFU/mL (Trial 3) and 8.36 x 10^8^ CFU/mL (Trial 4) after c.72 h post-inoculation, and then they both decreased up to the conclusion of the alcoholic fermentation (Figure 1C).

Then, the chemical composition of the obtained wines was assessed to evaluate the fermentative performances of the different starter formulations (FT-IR analysis) (Table 1).

Regarding residual sugars, higher values (1.50 and 1.66 g/L) were detected in Trials 2 and 4 respectively, while in the Trials 1 and 3 the concentrations of residual sugars were below 1.50 g/L. Comparing the four produce wines, we did not find significant differences among ethanol, total and volatile acidity, and tartaric acid detected amounts. As expected, the wine produced inoculating *L. plantarum* was denoted by the conversion of malic acid into lactic acid (2.64 g/L, Trial 3; 2 g/L, Trial 4). The highest concentrations of glycerol were detected in wines produced in the presence of *C. zemplinina* with 9.86 g/L and 9.13 g/L in Trials 2 and 4, respectively. The impact of non-*Saccharomyces* on spontaneous and inoculated malolactic fermentation has been receiving increasing attention [27,39,40]. Recently, two studies [41,42] have delved into the effect of *C. zemplinina* strains on both *O. oeni* and *L. plantarum* strains highlighting the relevance of inoculation time and underlining variable behaviours (stimulation, neutral influence, inhibiting) of MLF outcomes as a result of the different *C. zemplinina*/lactic acid bacterial couples of strains (in co-inoculation). The present results add a neutral effect of *C. zemplinina* 35NC1 on *L. plantarum* LP44, reinforcing the idea that the impact of the couples could be a strain-dependent property [42].

The four wines were then subjected to Gas Chromatographic-Mass Spectrometry (GC-MS), and the analysis allowed the identification of 32 different compounds belonging to five different families, i.e., alcohols, esters, acids, terpenes and lactones (Table 2). 

Considering the different classes of volatile organic compounds (VOCs), alcohols were quantitatively the largest group in the produced Negroamaro wines. These compounds are characterized by their steady and pungent smell/taste, and they are related to pleasant herbaceous notes, whereas higher concentrations (>400 mg/L) affect wine aroma [43]. Indeed, the maximum concentration of higher alcohols was found in Trial 4 (109.02 mg/L). Among alcohols, 3-methyl-1-butanol and 2-phenylethanol showed the highest concentrations ranging for the former from 18.43 mg/L (Trial 2) to 45.51 mg/L (Trial 4) and for the latter from 32.23 mg/L (Trial 2) to 59.50 mg/L (Trial 4). Of particular interest, in terms of statistical significance, is the fact that the higher concentration of 2-phenylethanol was found in the wine fermented by the mixture of the three starter cultures (Trial 4). In literature, *T. delbrueckii*/*S. cerevisiae* in co-culture led to an increase of 2-phenylethanol content in wine, conversely mixed-cultures with *C. zemplinina* and *S. cerevisiae* have shown a lower concentration of this compound, together with other alcohols [44,45]. Alcohols, such as 2-phenylethanol, are synthesized by the yeast from amino acids and/or simple sugars [46,47]. It is possible to speculate that the specific combination of *Lactobacillus plantarum*/*Saccharomyces cerevisiae*/*Candida zemplinina* strains might have boosted the concentration of a specific precursor.

Regarding esters, generally responsible for fruity notes [24,39], the higher concentrations were detected in wines fermented by mixed cultures characterized by the presence of LAB, i.e., Trial 3 and Trial 4. Several investigations have indicated that there is significant variability in the ester content of wines after the occurrence of the MLF [39,40,41,42,43,44,45,48], thus confirming the importance of malolactic bacteria strain selection for the wine quality improvement [49,50,51,52,53]. Ethyl esters of fatty acids are one of the most relevant groups of aroma compounds in wine [54], and the following were identified in our wines: Ethyl butanoate, ethyl hexanoate, ethyl lactate, ethyl octanoate, and decanoate. Additionally, among esters acetates, we identified isoamyl acetate and 2-phenethyl acetate. Ethyl lactate is associated with malolactic fermentation; indeed, it showed higher values 1.13 mg/mL and 1.16 mg/L, respectively in Trial 3 and 4, whose inoculum included *L. plantarum*. These data corroborate previous evidence about the positive action of LAB that can positively influence wine aroma by enhancing fruity, buttery, and creamy notes [42,49,50,55]. The 2-phenethylacetate, responsible for the odour of “banana” ranged from 0.04 mg/L (Trial 2) to 0.95 mg/L (Trial 4), thus supporting previous evidence that indicated the co-inoculation of *S. cerevisiae* with a selected non-*Saccharomyces* species as a tool to modulate the amount of phenethylacetate in produced wine [56].

Within the family of fatty acids, the amounts of 2-methyl hexanoic, hexanoic and octanoic acids were significantly higher in wine produced in the absence of *C. zemplinina* (Trial 1 and Trial 3). In contrast, the amount of 2-methyl propanoic, n-decanoic and 9-decenoic acids did not present statistically significant differences in the four obtained wines [52,53].

**Figure 2:**
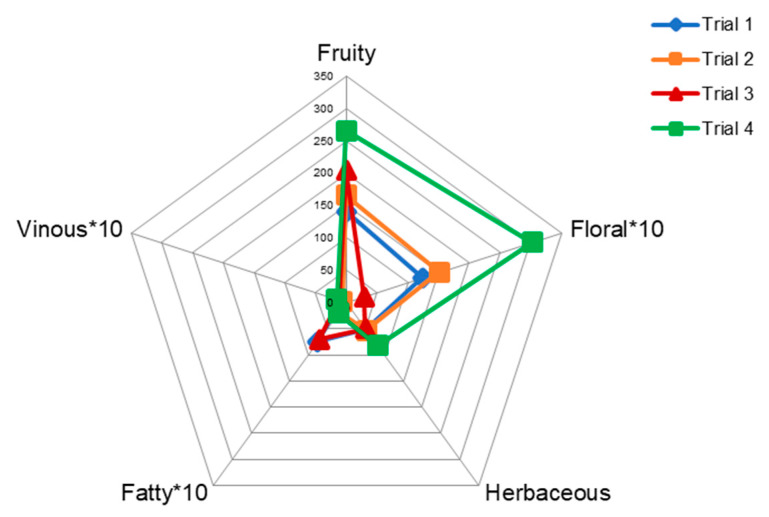
Mean value of the aromatic series calculated by adding the odour activity values of the compounds grouped in each one. The mean values of the aromatic series Vinous, Fatty, and Floral have been multiplied by a factor of 10.

Table 2 shows the perception threshold, descriptors, and odour series assigned for each compound quantified. The Odor Activity Value (OAV) of each volatile compound identified was determined, indicating the potential aroma contribution of individual molecules denoted by OAV > 1 [57,58]. However, it is important to underline that also compounds with lower OAVs can contribute to additive or synergic effects of the volatile components in the wine matrix [59]. Odorant series were obtained by grouping volatile aroma compounds having OAV > 1 and denoted with similar descriptors in one or several odorant series. In this respect, fruity, floral, herbaceous, fatty, and vinous odorant series were chosen for the description of Negroamaro aroma [60]. The sensorial radar plot as, simple and easy method to portrait the wine aroma profile, shows the odour profile of wines, obtained by the addition of OAVs values for their components belonging to the same odour descriptor class (Figure 2).

The fruity and floral series showed significant differences among the wines, in particular, higher values of OAV are associated with wine fermented by non-*Saccharomyces*, *Saccharomyces*, and *Lactobacillus* as result of high concentrations of esters and linalool. Besides, the fruity notes generated by esters are present in significant concentrations in wine produced in Trial 3. By contrast, this sample showed a lower OAV for floral notes. The fatty series associated with the volatile acids showed higher OAV, i.e., 74.90 and 70.18, in wines fermented by the monoculture of *S. cerevisiae* (Trial 1) and wine fermented by *S. cerevisiae* and *L. plantarum*, respectively (Trial 3). This representation suggests that the complexity of the wine can reflect the complexity of the starter cultures [3,44,61,62], when the latter relies on strains rigorously characterized and with well-tested compatibility. The issue is particularly relevant considering that an improved microbial diversity can be translated into an enhanced biotechnological potential. It is well-known the interest on selected non-*Saccharomyces* and malolactic strains to cope with specific technological issues in oenology, e.g., acidity improvement, alcohol reduction, and biocontrol activity [13,63,64,65,66]

### 3.2. Pilot-Scale Trials

To evaluate the oenological and fermentative performances of the newly defined mixed starter formulation, we tested the *C. zemplinina* 35NC1 strain combined with *S. cerevisiae* and *L. plantarum* for simultaneous inoculum of pilot-scale vinifications. The pilot-scale vinifications carried out as follow: Pilot A: *S. cerevisiae* NP103; Pilot B: co-inoculum of *S. cerevisiae* (NC103) + *L. plantarum* (LP44); Pilot C: co-inoculum of *S. cerevisiae* (NC103) and *C. zemplinina* (35NC1); Pilot D: co-inoculum of *C. zemplinina/S. cerevisiae/L. plantarum.* The four vinifications had a regular course, and they saw the completion of the alcoholic and malolactic fermentation processes seven days after the inoculation of the specific starter (data not shown). The main chemical parameters were evaluated by FT-IR analysis (Table 3).

Surprising, the pilot D, characterized by the presence of the three different microbial species, showed a slight but significant higher value of ethanol (13.50 g/L). In fact, in the literature, *C. zemplinina* has been proposed to achieve an alcohol reduction up to 0.7% (*v*/*v*) of ethanol in wine [17]. Our findings demonstrate a variability of this oenological trait in this specific non-*Saccharomyces*. For example, a similar variability has been reported for tolerance to ethanol in *C. zemplinina* [16,67]. No statistical differences were observed in volatile acidity among samples, ranging from 0.27 g/L (pilot A) to 0.38 g/L (pilot B). As expected, a decrease of malic acid, followed by an increase of lactic acid production were detected in pilot trials characterized by the presence of *L. plantarum* (Pilot B and D). According to the previous characterization [32], *L. plantarum* LP44 displayed particular fermentative abilities and tolerance of ethanol (up to 14%). Confirming the characterization of *C. zemplinina* 35NC1 in pure fermentation [23], the trials inoculated with this strain showed the highest concentrations of glycerol, i.e., 8.32 g/L in pilot C and 8.20 g/L in pilot D. A characteristic that can significantly enhance the body and fullness of produced wines [58,59,68]. The GC-MS analysis of volatile compounds in the wines produced by the pilot-scale vinifications allowed the identification of 27 molecules belonging to different chemical classes such as alcohols, esters, volatile acids, terpenes, sulphuric compounds, and volatile phenols (Table 4).

The wine from Pilot D showed higher concentrations of alcohols (118.77 mg/L), esters (18.92 mg/L), and terpenes (3.23 mg/L). High values of volatile acids were detected in Pilot B (5.56 mg/L) and C (4.54 mg/L). The wine produced with *S. cerevisiae* only (Pilot A) revealed a lower concentration of volatiles respect to the others and then a lower aromatic complexity. Among alcohols, the main compounds found were 3-methyl-1-butanol and phenylethanol. The first molecule ranged from 12.34 mg/L in pilot A to 40.28 mg/L in pilot D, and then in this latter being present at levels higher than its perception threshold (30 mg/L). Phenylethanol ranged from 21.20 mg/L in pilot C to 38.11 mg/L in pilot D. Both these volatile molecules belonging to higher alcohols family and are essential variables for differentiating between yeast strains because of their strict relation with yeast metabolism [60,61,69,70]. Among higher alcohols [62,63,64], phenylethanol is one of the key molecules of wine aroma being responsible for rose odour at a concentration above their odour threshold (14 mg/L) [71]. The ethyl esters of fatty acids, responsible for the “fruity” and “floral” sensory properties of wines, showed their lowest concentration in the pilot C wine (4.06 mg/L), while their maximum amount was found in the pilot D (18.62 mg/L). The wines from pilot A (2.14 mg/L) and pilot B (5.56 mg/L) vinifications showed the highest values of fatty acids, 2-methylpropanoic, 2-methylhexanoic, hexanoic, octanoic, and decanoic respect to the others. The pilot B and D wines showed the presence of both ethyl lactate (1.54 mg/L and 2.22 mg/L, respectively) and diethyl succinate (4.40 mg/L and 8.68 mg/L, respectively), they being the key molecules of malolactic fermentation (MLF) [8,39,63]. Moreover, the identification of specific VOCs in wines B and D (i.e., 3-hexen-1-ol (E), ethyl lactate and hydroxy diethyl malate) corroborates the previous indication about the capacity of *L. plantarum* to produce by its own or to modulate the yeast- specific synthesis of aromatic compounds [28,63].

**Figure 3:**
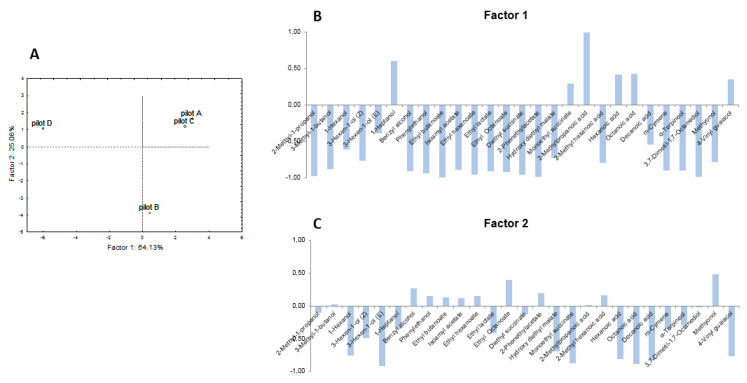
Score plot (**A**) and loading plots of the first (**B**) and second (**C**) principal components after PCA of volatile compounds detected in the for wines produced by pilot-scale vinification.

To evaluate the overall effect of the different mixed starter formulations on the volatile profiles of produce wines, the principal component analysis (PCA) was carried out (Figure 3).

The first two principal components explained the 89.19% of the variance in the data set (PC1 = 64.13%, PC2 = 25.06%). As shown in Figure 3, the PC1 was correlated with 3-methyl-1-butanol, 2-methyl-1-propanol, 3-hexen-1-ol (Z), 1-heptanol, benzylalcohol, phenylethanol, ethyl butanoate, isoamyl acetate, ethyl hexanoate, ethyl lactate, ethyl octanoate, diethyl succinate, phenyl acetate, hydroxy diethyl malate, 2-methylhexanoic acid, cymene, α-terpineol, 3,7-dimethyl-1,7-octanediol, methyonol, and positively with 2-methylpropanoic acid (correlation 0.99; Figure 3).

The second component PC2 was negatively correlated with 1-hexanol, 3-hexen-1-ol (E), monoethyl succinate, hexanoic, octanoic, and decanoic acids and 4-vinylguaiacol (correlation higher than 0.75; Figure 3). Three groups were observed, i.e. the wine fermented by *Saccharomyces* with *Lactobacillus* (Pilot B) (bottom right quadrant of PC2), the wines that did not undergo malolactic fermentation (Pilot A and C) (top right quadrant of PC1), finally, the wine that underwent MFL (Pilot D) characterized by the activity of three microorganisms (top left quadrant of PC1). The first PCA (64.13%) was negatively correlated with the wine sample D (*C. zemplinina* + *S. cerevisiae + L. plantarum)* and positively correlated with samples A (*S. cerevisiae*) and C (*C. zemplinina* + *S. cerevisiae*). In comparison, the second PCA (25.06%) is linked to sample B (*S. cerevisiae+ L. plantarum)*. The wine samples A, C, and D described by PC1 show qualitatively similar but quantitatively different volatile profiles except for some volatiles correlated with malolactic fermentation such as hydroxy diethyl malate, ethyl lactate, and diethyl succinate. Higher values of volatile molecules belonging to esters and alcohols classes associated with PC1 were detected in wine D, while the wine B characterized by the double activity of *Saccharomyces* and *Lactobacillus,* was associated with PC2 (25.06%).

### 3.3. Industrial-Scale Vinifications

The industrial-scale vinifications were carried out in an industrial winery cellar of Salento (Apulia, Southern Italy) during the vintage 2017 and 2018 by fermenting 10.000 L of Negroamaro grape must. Together with the experimental trial, three similar control vinifications were performed by the separately inoculating a different commercial yeast preparation, they being routinely employed in the winery, followed by the addition at the end of the alcoholic fermentation of a commercial malolactic starter. The fermentation processes inoculated with the mixed starter formulation have taken place regularly during both the vintages. The sugars and malic acid consumption were respectively completed in 5 and 6 days for the 2017 production and 6 and 7 days for the 2018 vinification (Supplementary Materials Figure S1). The results of chemical analysis of the wine obtained by mixed inoculum (Mixstart_year) are shown in Table 5, in comparison to the same must fermented or by the sequential inoculation of three different commercial yeast preparations followed by the addition of a commercial malolactic starter at the end of the alcoholic fermentation (Comm_year).

Regarding the content of ethanol, total and volatile acidity, statistical differences among the samples were not found. On the other hand, the amount of residual sugars detected in the wine produced with the mixed starter formulation, respectively 0.04 g/L for the Mixstart_2017 and 0.20 g/L for the Mixstart_2018 wines, were statistically different from those found in the wines produced with the commercial starters (Table 5). All produced wines underwent malolactic fermentation, and the highest values of lactic acid were observed in wines fermented by the mixed starter formulation, i.e., 2.83 g/L for Mixstart_2017 and 2.65 g/L for Mixstart_2018. 

**Figure 4:**
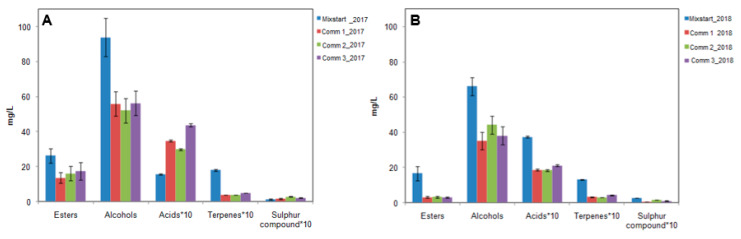
Volatile compounds classes quantified in wines produced at the industrial scale during the vintages 2017 (**A**) and 2018 (**B**). The values of the classes Acids, Terpenes, and Sulphur compounds have been multiplied by a factor of 10.

To evaluate the impact of the mixed culture of wine organoleptic characteristics, also the volatile fraction of wines produced at the industrial-scale was evaluated using the solid-phase extraction coupled to GC-MS (Supplementary Materials Tables S1 and S2). The volatile molecules concentrations were quantified in the wines produced during the vintage 2017 and 2018, and they were grouped by chemical class (Figure 4). The results showed that the wines produced with the novel mixed starter had higher concentrations of volatile compounds, such as alcohols, esters and terpenes, molecules that give the highest contribution to the overall wine aroma. In terms of reproducibility of the results, it is possible to underline a certain homogeneity of the trends across the two vintages for the classes of esters, alcohols, and terpenes. The class of acids represents an exception.

**Figure 5:**
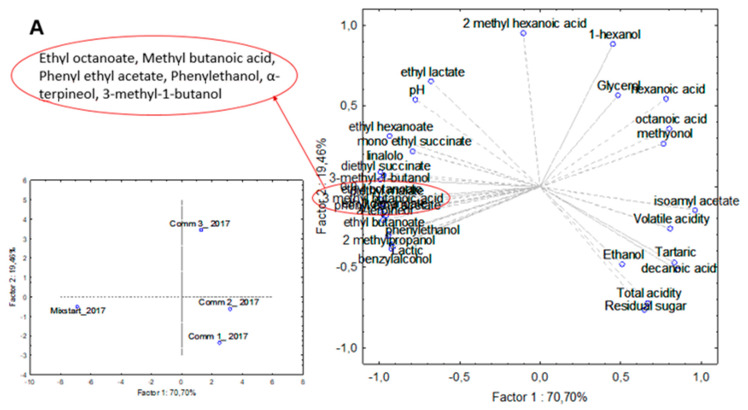

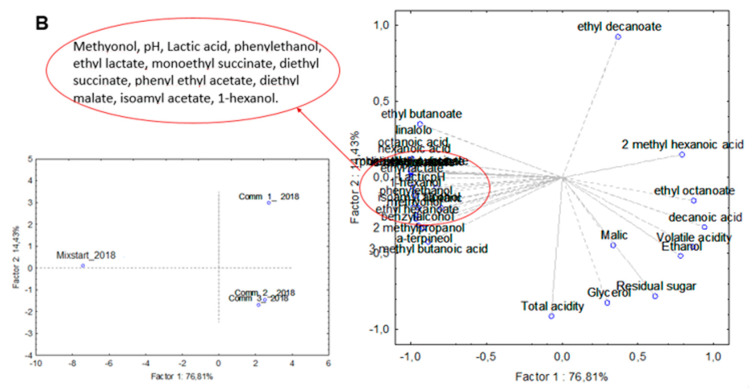
Principal Component Analysis (PCA) performed employing the data obtained by the chemical analysis of the wines produced at the industrial scale during the vintages 2017 (**A**) and 2018 (**B**).

The above data were statistically evaluated using to the PCA, in order to evaluate the correlations between starters formulation and volatile molecules identified and main chemical parameters (Figure 5)

As shown in Figure 4A,B, for data associated with vintage 2017 the two first principal components explained about 90.16% of the total variance, while for data associated with vintage 2018 the amount of the two first principal components was 91.24%. Regarding the wines from the vintage 2017 (Figure 5A), the first PCA dimension (70.70% of explained variance) discriminates the wine produced with the novel mixed starter formulation. The Startmix_2017 lies on the negative semi-axis of the first component, and it differed from the other three commercial wines, since the high content of ethyl octanoate, methyl butanoic acid, phenylethyl acetate, phenylethanol, α-terpineol, linalool, diethyl succinate, and 3-methyl-1-butanol. A significant presence of isoamyl acetate, volatile acidity, methyonol and octanoic acid positively correlated to PC1 semi-axis (70.70% of variance), characterized Comm 2_2017 wine. In comparison, the second dimension (19.46% of explained variance) discriminates these two last commercial wines, Comm 1_2017 lying on negative semi-axis of PC2. The Comm 3_2017 wine lay on positive semi-axis of the same PC and 2-Methyl hexanoic acid, ethyl lactate, and 1-hexanol, for their high presence, contribute to its discrimination. Concerning the wines produced during the vintage 2018, Figure 5B shows the scores scatter plot and the corresponding loadings plot for the first two PCs. The Startmix_2018 wine was associated to negative semi-axis negative of PC1 (76.81% of variance) characterized by the presence of most of the volatiles molecule identified such as phenylethanol, ethyl lactate, monoethyl succinate, diethyl succinate, phenyl ethyl acetate, diethyl malate, isoamyl acetate, and 1-hexanol and then they seemingly influence the complexity of the Startmix_2018 wine aroma profile. Commercial wines cluster at positive (Comm 1_2018) and negative (Comm 2_2018 and Comm 3_2018) PC2 (14.43% of variance). The Comm 2_2018 and Comm 3_2018 wines contained high relative correlations mainly of total acidity, residual sugars, and glycerol in respect to the Comm 1_2018 wine. In both vintages, the wines fermented with the mixed starter mixture showed a high correlation with the most important classes of volatile molecules such as alcohols, esters, terpenes showing a more complex volatile profile. The wines CNR_2017 and CNR_2018 showed an increase of phenylethanol, 3-methyl-1-butanol in concentrations above their odour threshold and then higher values of terpenes such as linalool and α-terpineol associated with floral notes, and esters linked to malolactic fermentation such as ethyl lactate, diethyl and mono ethyl succinate.

Interaction with yeasts can be from stimulatory, to neutral or inhibitory, depending on the secretion of nutrients by yeasts and on their capacity to synthesize metabolites able to affect LAB growth [37]. The data obtained indicated that the co-cultivation with the tested strains, also at the industrial scale, did not cause negative effects on the physiological behavior of the three employed species. Reported findings confirmed that the employment of *S. cerevisiae*/LAB mixed inoculums for the controlling of the MLF can be uninfluenced by the addition of a non-*Saccharomyces* starter strain, resulting in a positive effect on fermentation time and aromatic composition of wine [41]. Volatile organic compounds derived from yeasts- and LAB-promoted fermentation have a pivotal importance in determining the aroma profile of wines [63]. 

In the present study, the addition of *L. plantarum* strain LP44, together with the yeast inoculation, caused significant variations in the volatile composition of Negroamaro wines. Total volatile and ester concentrations generally increased when *L. plantarum* was added to the inoculum, as indicated by the results of the GC-MS of the wines produced in the lab- (Trial 4; Table 2), pilot- (Pilot D; Table 4), and industrial-scale (Mixstart_2017 and Mixstart_2018; Supplementary Materials Tables S1 and S2, Figure 4) vinifications. In agreement with previous evidence [8], the above wines produced by co-inoculation of the three microbial species were denoted by enhanced fruity notes, it is likely to be explained by the metabolic interactions between yeasts and bacteria [8,48,72,73]. This exploitation of microbial diversity in the production of typical wines with unique pronounced sensory quality is relevant to provide a robust (and sustainable) alternative to the instances for a return to spontaneous fermentation for the differentiation of traditional/artisanal fermented foods and beverages (the latter being a trend that poses risks for human health and the quality of fermentative processes) [74,75,76,77].

**Figure 6:**
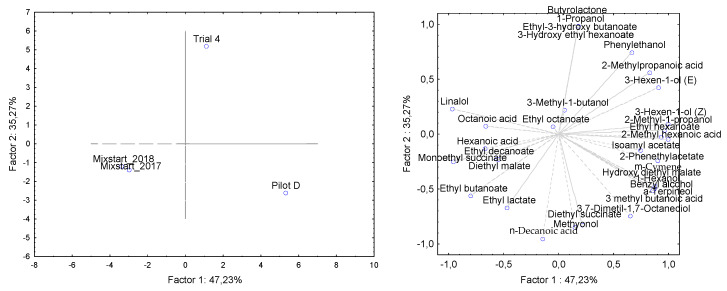
Principal component analysis (PCA) scores and loadings for secondary compounds identified in wines produced at the lab-scale (Trial 4), pilot-scale (Pilot D), and industrial-scale (Mixstart 2017–2018).

Due to the complexity and the variability of a large number of environmental, chemical, technological, and biological factors which impact on the final quality of the wine, research in oenology tends to rely on small-scale productions, allowing well-controlled conditions and adequate reproducibility [78]. Nevertheless, scientists in the field continue to explore the effect of scale comparing the differences between small-scale and industrial-scale [78,79,80,81]. Considering the relevance of evaluating new mixed starter cultures at different scales, it appears interesting to provide a comparative representation among the diverse volumes of vinification this study explored. With this purpose, all the simultaneous inoculation of non-*Saccharomyces*, *S. cerevisiae*, and *L. plantarum* at the different scales (lab-scale, Trial 4; pilot-scale, Pilot D; industrial-scale, Mixstart_2017; industrial-scale, Mixstart_2018) were visualized through a principal component analysis (PCA) (Figure 6).

The distribution of variances indicates a clear separation among lab-scale, pilot-scale, and industrial-scale trials, suggesting that VOCs differently contribute to the chemical diversity of produced wines. In effect, observing the single compounds, several trends can be highlighted. Ten molecules were found only in one of the scales (lab-scale: 1-propanol, 1-butanol, 3-methyl-pentanol, ethyl-3-hydroxy butanoate, 3-hydroxy ethyl hexanoate, butyrolactone; pilot-scale: hydroxy diethyl malate, m-cymene, α-terpineol; industrial-scale: 3-methyl butanoic acid). Seven compounds were absent in one of the tested volumes of vinification (lab-scale: methyonol; pilot-scale: ethyl decanoate, diethyl malate, monoethyl succinate, linalol; industrial-scale: 3-hexen-1-ol (Z), 3-hexen-1-ol (E)). For several other chemicals, the concentrations changed as a function of the scale (e.g., 1-hexanol, benzyl alcohol, phenylethanol, ethyl lactate, 2-phenethylacetate, hexanoic acid). This molecular variability underlines the importance to investigate the oenological and biological consequences of a variable winemaking scales in the assessment of the impact of new starter cultures in terms of VOCs contribution.

## 4. Conclusions

Our results add a further piece to the puzzle of microbial management of autochthonous oenological resources of Apulian region [1,25,26,32,33,36,82,83,84,85,86,87,88,89], the second Italian region for wine production, (particularly for red and rosé wines) [37]. The obtained evidence allow consideration of this microbial mixture as a valid solution to enhance the peculiar features of typical regional wines, contrasting the tendency of a return to spontaneous fermentation. To the best of our knowledge, this work first tested the utilization of a mixed starter culture including species belonging to non-*Saccharomyces* (*C. zemplinina*), *Saccharomyces cerevisiae*, and *Lactobacillus plantarum* for production of wine at the industrial scale. The results underline the shifts of “volatome” addressable to the different combinations of the strains, with particular attention to the simultaneous inoculation of the three species, confirming that the complexity of the wine can reflect the complexity of the starter cultures. Finally, this work highlights the relevance of a complete-scale assessment (up to the real commercial-scale) to define the contribution of a multi-species starter culture in terms of VOCs diversity. 

## Figures and Tables

**Table 1:** Concentration of major chemical compounds in wines obtained with selected strains.

Trial	Ethanol	Sugars	TA	VA	pH	Malic	Lactic	Glycerol
1	12.14 ± 1.12	1.13 ± 0.20	9.38 ± 1.24	0.22 ± 0.02	3.00 ± 0.15	2.17 ^b^ ± 0.61	0.01 ^a^ ± 0.008	8.17 ± 1.40
2	12.23 ± 1.20	1.50 ± 0.49	9.14 ± 1.11	0.30 ± 0.01	3.08 ± 0.05	2.00 ^b^ ± 0.04	nd	9.86 ± 1.42
3	12.65 ± 1.44	1.36 ± 0.16	9.36 ± 1.17	0.23 ± 0.07	3.11 ± 0.21	0.02 ^a^ ± 0.00	2.64 ^b^ ± 0.33	8.55 ± 1.42
4	12.00 ± 1.25	1.66 ± 0.21	9.29 ± 1.33	0.29 ± 0.06	3.09 ± 0.15	0.02 ^a^ ± 0.01	2.00 ^b^ ± 0.25	9.13 ± 1.34

TA, total acidity. VA, volatile acidity. Values are expressed in g/L. The ethanol concentration is expressed in g/100 mL. Results are the mean of three replicates; the standard deviation values (±) are indicated. Different letters in the column denote significant differences between different inoculum trials, at *p* < 0.05; nd: not determined.

**Table 2:** The concentration of volatile compounds determined in the four wines obtained by the lab-scale vinifications.

Compounds	Trial 1	Trial 2	Trial 3	Trial 4	Odour Thresh Old	Sensory Notes	Odorant Series
**Alcohols**							
1-Propanol	0.20 ^a^ ± 0.04	0.15 ^a^ ± 0.05	nd	0.31 ^a^ ± 0.07	306	Ripe fruit, alcohol	
2-Methyl-1-propanol	1.85 ^a^ ± 0.30	1.56 ^a^ ± 0.35	1.47 ^a^ ± 0.24	2.84 ^a^ ± 0.65	0.2	Bitter, green, harsh	1
1-Butanol	0.07 ^a^ ± 0.02	nd	0.06 ^a^ ± 0.02	0.07 ^a^ ± 0.02	150		
3-Methyl-1-butanol	26.82 ^a^ ± 5.48	18.43 ^a^ ± 4.90	32.23 ^a^ ± 4.67	45.51 ^a^ ± 8.97	30	Vinous fusel alcohol	2
3-Methyl-pentanol	0.55 ^a^ ± 0.12	0.76 ^a^ ± 0.13	0.11 ^a^ ± 0.03	0.95 ^a^ ± 0.23	1.1	Pungent, cocoa, wine-like	
1-Hexanol	0.47 ^a^ ± 0.07	0.34 ^a^ ± 0.06	0.41 ^a^ ± 0.06	0.56 ^a^ ± 0.11	8	Green	
3-Hexen-1-ol (Z)	0.02 ^a^ ± 0.005	nd	nd	0.03 ^a^ ± 0.005	1	Herbaceous, green	
3-Hexen-1-ol (E)	0.07 ^a^ ± 0.005	0.07 ^a^ ± 0.02	0.07 ^a^ ± 0.03	0.10 ^a^ ± 0.02	15	Green	
Benzyl alcohol	0.07 ^a^ ± 0.02	0.07 ^a^ ± 0.03	0.08 ^a^ ± 0.02	0.10^a^ ± 0.02	900	Burning taste	
Phenylethanol	39.07 ^a^ ± 7.11	33.23 ^a^ ± 4.58	40.57 ^a^ ± 7.21	59.50 ^b^ ± 9.55	14	Rose floral	3
**Esters**							
Ethyl butanoate	0.25 ^a^ ± 0.02	0.38 ^a^ ± 0.08	0.55 ^a^ ± 0.11	0.72 ^a^ ± 0.13	0.02	Fruity apple	4
Isoamyl acetate	0.26 ^a^ ± 0.06	0.28 ^a^ ± 0.02	0.55 ^a^ ± 0.03	0.73^a^ ± 0.13	0.03	Banana	4
Ethyl hexanoate	0.59 ^a^ ± 0.02	0.64 ^a^ ± 0.02	0.60 ^a^ ± 0.03	0.95 ^a^ ± 0.12	0.014	Green apple, anise	1,4
Ethyl lactate	0.14 ^a^ ± 0.03	0.08 ^a^ ± 0.02	1.13 ^b^ ± 0.03	1.16 ^b^ ± 0.03	150		
Ethyl octanoate	0.38 ^a^ ± 0.12	0.46 ^a^ ± 0.14	0.57 ^a^ ± 0.13	0.67 ^a^ ± 0.14	0.005	Sweet, fruity, fresh	4
Ethyl-3-hydroxy butanoate	0.04 ^a^ ± 0.005	0.03 ^a^ ± 0.006	0.03 ^a^ ± 0.004	0.032 ^a^ ± 0.005	1	Fruity, grape	4
Ethyl decanoate	0.67 ^a^ ± 0.12	0.55 ^a^ ± 0.10	0.76 ^a^ ± 0.25	1.10 ^a^ ± 0.22	0.2	Fruity, sweet, grape	4
Diethyl succinate	0.31 ^a^ ± 0.04	0.11 ^a^ ± 0.05	0.22 ^a^ ± 0.006	0.13 ^a^ ± 0.03	6	Wine	
3-Hydroxy ethyl hexanoate	nd	0.017 ^a^ ± 0.005	nd	0.028 ^a^ ± 0.04	NA		
1,3-propandiol acetate	0.07 ^a^ ± 0.02	nd	0.09 ^a^ ± 0.02	nd	NA		
2-Phenethylacetate	0.13 ^a^ ± 0.05	0.33 ^a^ ± 0.02	0.45 ^a^ ± 0.06	0.95^a^ ± 0.15	0.25	Fruity	4
Diethyl malate	0.06 ^a^ ± 0.02	0.06 ^a^ ± 0.02	0.07 ^a^ ± 0.02	0.10 ^a^ ± 0.03	10	Fruity	
Monoethyl succinate	1.93 ^b^ ± 0.45	0.66 ^a^ ± 0.07	1.44 ^b^ ± 0.54	1.61 ^b^ ± 0.44	NA		
**Terpenes**							
Linalol	0.24 ^a^ ± 0.06	0.32 ^a^ ± 0.10	nd	0.65 ^a^ ± 0.20	0.025	Floreal	3
**Volatile acids**							
2-Methylpropanoic acid	0.20 ^a^ ± 0.05	0.13 ^a^ ± 0.04	0.24 ^a^ ± 0.05	0.25 ^a^ ± 0.06	NA		
Butanoic acid	nd	nd	0.09 ± 0.02	nd	2.2	Cheesy	
2-Methyl hexanoic acid	0.92 ^a^ ± 0.11	0.24 ^a^ ± 0.06	0.90 ^a^ ± 0.16	0.34 ^a^ ± 0.05			
Hexanoic acid	1.23 ^b^ ± 0.06	0.35 ^a^ ± 0.08	1.15 ^b^ ± 0.35	0.40 ^a^ ± 0.06	0.42	Fatty acid, cheese	5
Octanoic acid	2.28 ^b^ ± 0.55	0.55 ^a^ ± 0.16	2.14 ^b^ ± 0.07	0.54 ^a^ ± 0.10	0.5	Fatty acid, cheese	5
*n*-Decanoic acid	0.62 ^a^ ± 0.07	nd	0.41 ^a^ ± 0.07	nd	1		
9-Decenoic acid	0.69 ^a^ ± 0.18	nd	0.50 ^a^ ± 0.06	nd	NA		
**Lactones**							
Butyrolactone	0.25 ^a^ ± 0.03	0.17 ^a^ ± 0.03	nd	0.33 ^a^ ± 0.07	NA		

Values are expressed in mg/L. They are the mean of three replicates, and the standard deviation values (±) are indicated. Different letters in the row denote significant differences between yeast strains, at *p* < 0.05. Odorant series: 1, Herbaceous; 2, Vinous; 3 Floral; 4, Fruit; 5, Fatty. We reported the association with odorant series only for molecules with OAV > 1. Odor threshold and sensory notes are reported according to Tufariello et al. [43]. nd, not determined; NA, not available.

**Table 3:** Concentration of major chemical compounds in wines obtained by the for pilot-scale vinifications.

Trials	Ethanol	Sugars	TA	VA	pH	Malic	Lactic	Glycerol
Pilot A	12.13 ^a^ ± 1.61	0.27 ^a^ ± 0.09	6.65 ^b^ ± 0.34	0.27 ^a^ ± 0.01	3.38 ^a^ ± 0.12	1.74 ^a^ ± 0.027	0.13 ^a^ ± 0.09	6.21 ^a^ ± 1.41
Pilot B	12.22 ^a^ ± 1.23	1.12 ^b^ ± 0.06	4.92 ^a^ ± 0.62	0.38 ^a^ ± 0.02	3.53 ^a^ ± 0.14	nd	1.38 ^b^ ± 0.16	6.58 ^a^ ± 1.42
Pilot C	12.34 ^a^ ± 1.11	0.71 ^a^ ± 0.09	5.20 ^a^ ± 0.21	0.30 ^a^ ± 0.02	3.52 ^a^ ± 0.11	1.75 ^a^ ± 0.027	0.14 ^a^ ± 0.04	8.32 ^b^ ± 1.25
Pilot D	13.50 ^b^ ± 1.80	0.11 ^a^ ± 0.04	5.12 ^a^ ± 0.16	0.33 ^a^ ± 0.01	3.55 ^a^ ± 0.13	nd	1.78 ^b^ ± 0.12	8.20 ^b^ ± 1.45

TA, total acidity. VA, volatile acidity. Values are expressed in g/L. The ethanol concentration is expressed in g/100 mL. Results are the mean of three replicates; the standard deviation values (±) are indicated. Different letters in the column denote significant differences between different inoculum trials, at *p* < 0.05; nd: not detection.

**Table 4:** Volatile compounds identified and quantified in the four wines produced in the pilot scale.

Compounds	Pilot A	Pilot B	Pilot C	Pilot D
**Alcohols**				
2-Methyl-1-propanol	3.17 ^a^ ± 0.04	3.70 ^a^ ± 0.05	2.90 ^a^ ± 0.025	4.80 ^a^ ± 0.34
3-Methyl-1-butanol	12.34 ^a^ ± 3.55	33.67 ^a^ ± 4.76	26.54 ^a^ ± 4.55	40.28 ^a^ ± 6.11
1-Hexanol	0.90 ^a^ ± 0.11	2.73 ^b^ ± 0.44	0.48 ^a^ ± 0.07	2.20 ^b^ ± 0.47
3-Hexen-1-ol (Z)	0.020 ^a^ ± 0.007	0.045 ^a^ ± 0.005	nd	0.05 ^a^ ± 0.02
3-Hexen-1-ol (E)	nd	0.22 ^a^ ± 0.06	nd	0.10 ^a^ ± 0.03
1-Heptanol	0.04 ^a^ ± 0.02	0.15 ^b^ ± 0.04	0.21 ^b^ ± 0.07	nd
Benzyl alcohol	8.21 ^a^ ± 2.10	21.67 ^b^ ± 3.93	17.45 ^b^ ± 4.10	33.23 ^b^ ± 7.36
Phenylethanol	16.20 ^a^ ± 4.43	30.70 ^b^ ± 5.33	21.20 ^a^ ± 4.38	48.11 ^b^ ± 4.17
**Esters**				
Ethyl ^b^ utanoate	0.45 ^a^ ± 0.11	0.52 ^a^ ± 0.16	0.45 ^a^ ± 0.12	0.95 ^a^ ± 0.14
Isoamyl acetate	0.18 ^a^ ± 0.05	0.50 ^a^ ± 0.06	0.67 ^a^ ± 0.13	1.20 ^a^ ± 0.26
Ethyl hexanoate	0.19 ^a^ ± 0.05	0.49 ^a^ ± 0.05	0.58 ^a^ ± 0.11	1.50 ^a^ ± 0.12
Ethyl lactate	nd	1.54 ^a^ ± 0.15	nd	2.22 ^a^ ± 0.58
Ethyl Octanoate	0.27 ^a^ ± 0.06	0.22 ^a^ ± 0.06	0.25 ^a^ ± 0.06	0.52 ^a^ ± 0.15
Diethyl succinate	2.38 ^ab^ ± 0.06	4.40 ^b^ ± 0.55	0.62 ^a^ ± 0.06	8.68 ^c^ ± 2.58
2-Phenethylacetate	0.06 ^a^ ± 0.02	0.22 ^a^ ± 0.06	0.05 ^a^ ± 0.02	2.15 ^b^ ± 0.56
Hydroxy diethyl malate	nd	1.56 ^a^ ± 0.21	nd	1.40 ^a^ ± 0.60
**Volatile acids**				
2-Methylpropanoic acid	0.45 ^a^ ± 0.11	0.37 ^a^ ± 0.05	0.41 ^a^ ± 0.11	0.20 ^a^ ± 0.05
2-Methyl hexanoic acid	nd	0.22 ^a^ ± 0.06	0.43 ^a^ ± 0.01	0.68 ^a^ ± 0.11
Hexanoic acid	0.48 ^a^ ± 0.07	0.73 ^a^ ± 0.45	0.27 ^a^ ± 0.06	0.20 ^a^ ± 0.05
Octanoic acid	1.09 ^a^ ± 0.34	1.42 ^a^ ± 0.74	0.72 ^a^ ± 0.12	0.17 ^a^ ± 0.04
Decanoic acid	0.12 ^a^ ± 0.03	0.82 ^a^ ± 0.12	0.11 ^a^ ± 0.03	0.56 ^a^ ± 0.11
**Terpenes**				
*m*-Cymene	0.12 ^a^ ± 0.05	0.42 ^a^ ± 0.05	0.28 ^a^ ± 0.10	0.56 ^a^ ± 0.06
α-Terpineol	0.11 ^a^ ± 0.04	0.34 ^a^ ± 0.05	0.25 ^a^ ± 0.04	0.47 ^a^ ± 0.02
3,7-Dimetil-1,7-Octanediol	1.11 ^a^ ± 0.03	1.37 ^a^ ± 0.11	0.95 ^a^ ± 0.11	2.20 ^a^ ± 0.65
**Sulphur compound**				
Methyonol	0.13 ^a^ ± 0.01	nd	nd	0.28 ^a^ ± 0.06
**Volatile phenol**				
4-Vinyl guaiacol	nd	0.31 ^a^ ± 0.06	0.22 ^a^ ± 0.06	nd

Values are expressed in mg/L. They are the mean of three replicates and the standard deviation values (±) are indicated. Different letters in the row denote significant differences between yeast strains, at *p* < 0.05.

**Table 5:** The concentration of major chemical compounds in Negroamaro wines produced in the industrial scale during the vintage 2017 and 2018, by separately inoculating the same grape must with the mixed starter formulation (Mixstart_year) or by the sequential inoculation commercial yeast and malolactic starter (Comm_year).

	Ethanol	Sugar	TA	VA	pH	Malic	Lactic	Glycerol
**Mixstart_2017**	11.97 ^a^ ± 0.55	0.04 ^a^ ± 0.01	6.32 ^a^ ± 0.20	0.34 ^a^ ± 0.05	3.64 ^a^ ± 0.23	nd	2.83 ^b^ ± 0.05	9.79 ^a^ ± 0.56
**Comm 1_ 2017**	12.06 ^a^ ± 1.54	0.86^c^ ± 0.14	6.86 ^a^ ± 0.13	0.41 ^a^ ± 0.06	3.37 ^a^ ± 0.21	nd	1.70 ^a^ ± 0.21	9.66 ^a^ ± 0.54
**Comm 2_ 2017**	12.30 ^a^ ± 2.10	0.65 ^b^ ± 0.11	6.64 ^a^ ± 1.10	0.49 ^a^ ± 0.05	3.49 ^a^ ± 0.43	nd	1.41 ^a^ ± 0.05	10.66 ^a^ ± 1.10
**Comm 3_ 2017**	11.92 ^a^ ± 1.11	nd	6.35 ^a^ ± 0.54	0.38 ^a^ ± 0.04	3.56 ^a^ ± 0.23	nd	1.22 ^a^ ± 0.04	10.44 ^a^ ± 0.17
**Mixstart_2018**	13.69 ^a^ ± 2.13	0.20 ^a^ ± 0.02	6.51 ^a^ ± 0.23	0.23 ^a^ ± 0.06	3.68 ^a^ ± 0.16	nd	2.65 ^b^ ± 0.21	10.48 ^a^ ± 0.22
**Comm 1_ 2018**	13.40 ^a^ ± 0.23	0.99 ^b^ ± 0.20	6.17 ^a^ ± 1.55	0.31 ^a^ ± 0.05	3.62 ^a^ ± 0.05	nd	1.75 ^a^ ± 0.12	10.18 ^a^ ± 1.45
**Comm 2_ 2018**	13.80 ^a^ ± 0.33	0.52 ^c^ ± 0.05	6.75 ^a^ ± 1.30	0.35 ^a^ ± 0.05	3.60 ^a^ ± 0.30	0.26 ± 0.07	1.73 ^a^ ± 0.11	10.92 ^a^ ± 0.55
**Comm 3_ 2018**	13.44 ^a^ ± 0.55	0.67 ^c^ ± 0.11	6.54 ^a^ ± 0.54	0.38 ^a^ ± 0.05	3.65 ^a^ ± 0.43	nd	1.94 ^a^ ± 0.14	11.80 ^a^ ± 0.50

TA, total acidity. VA, volatile acidity. Values are expressed in g/L. The ethanol concentration is expressed as g/100 mL. Results are the mean of three replicates; the standard deviation values (±) are indicated. Different letters in the row denote significant differences between different inoculum trials, at *p* < 0.05; nd: not determined.

## Supplementary Materials

The following are available online at https://www.mdpi.com/2076-2607/8/5/726/s1, Supplementary File 1: PDF-Document.
