# Supplementary material for: Effect of Co-Inoculation of Candida zemplinina, Saccharomyces cerevisiae and Lactobacillus plantarum for the Industrial Production of Negroamaro Wine in Apulia (Southern Italy)
Source: Microorganisms. 2020 May 13;8(5):726. doi: 10.3390/microorganisms8050726 (PMC7285497; doi:10.3390/microorganisms8050726)
Supplement: Supplementary file 1 [file microorganisms-08-00726-s001.pdf]

A

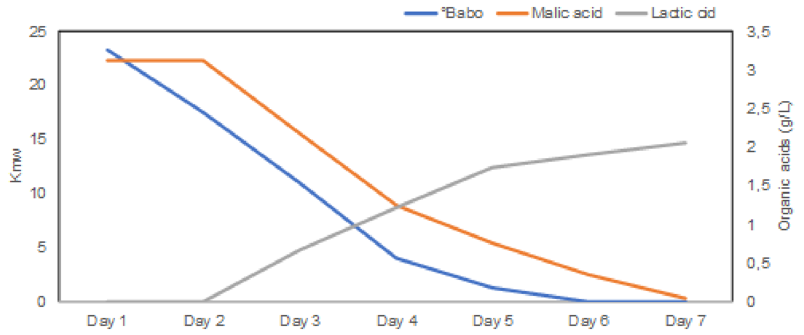

B

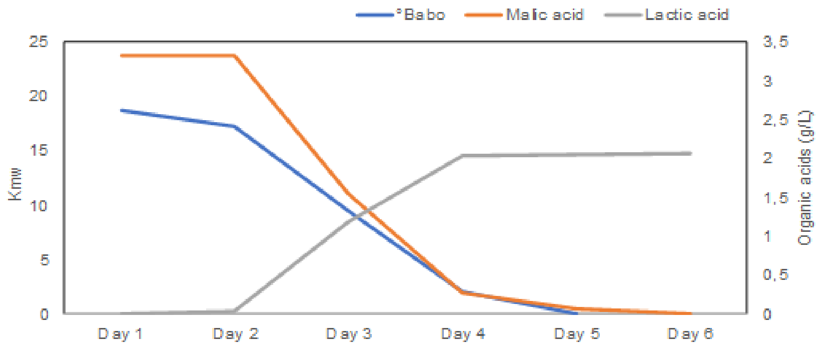

Figure S1. Production of lactic acid and consumption of sugars and malic acid monitored during the industrial-scale vinification inoculated with by the mixed inoculum (Mixstart) during the vintage 2017 (A) and 2018 (B).

Table S1. The concentration of volatile compounds determined in the four wines obtained by the industrial-scale vinifications during the vintage 2017. The vinifications were carried out by separately inoculating in the same grape must with the mixed starter formulation (Mix_start) or by the sequential inoculation of three different commercial yeast preparations followed by the addition of a commercial malolactic starter at the end of the alcoholic fermentation (Comm).

Compounds	Mixstart_2017	Comm 1_ 2017	Comm 2_ 2017	Comm 3_ 2017
**Esters**				
ethyl butanoate	1.56±0.34	1.29±0.15	1.16±0.04	1.20±0.04
isoamyl acetate	0.21±0.06	0.40±0.16	0.46±0.11	0.35±0.11
ethyl hexanoate	0.25±0.11	0.18±0.05	0.17±0.04	0.21±0.05
ethyl lactate	3.87±0.45	2.52±0.45	2.11±0.16	3.94±0.17
ethyl octanoate	1.27±0.15	0.58±0.16	0.57±0.21	0.62±0.17
ethyl decanoate	1.48±0.11	0.95±0.27	0.76±0.28	0.88±0.25
diethyl succinate	8.45±2.56	4.50±0.95	5.26±0.73	5.44±0.65
phenyl ethyl acetate	0.94±0.15	0.36±0.06	0.44±0.16	0.35±0.05
diethyl malate	1.76±0.33	0.16±0.06	0.37±0.07	0.25±0.07
mono ethyl succinate	6.47±1.67	2.68±0.85	4.83±0.94	4.33±0.76
**Alcohols**				
2 methyl-1-propanol	3.56±0.45	1.90±0.27	1.15±0.12	1.13±0.06
3-methyl-1-butanol	52.98±7.10	28.27±5.17	29.71±4.10	33.14±5.17
1-hexanol	0.43±0.05	0.45±0.05	0.69±0.15	0.94±0.17
methyonol	0.11±0.04	0.16±0.04	0.29±0.06	0.22±0.04
benzylalcohol	0.76±0.21	0.35±0.11	0.12±0.03	0.13±0.03
phenylethanol	36.04±5.60	24.77±5.10	20.14±4.19	20.74±3.80
**Acids**				
3 methyl butanoic acid	0.036±0.012	nd	nd	nd
2 methyl hexanoic acid	0.096±0.024	nd	nd	0.34±0.06
hexanoic acid	0.41±0.11	1.41±0.15	1.28±0.07	2.01±0.32
octanoic acid	0.36±0.06	1.13±0.17	0.83±0.13	1.28±0.27
decanoic acid	0.65±0.21	0.93±0.21	0.88±0.22	0.74±0.06
**Terpenes**				
linalolo	0.94±0.20	0.16±0.05	0.22±0.08	0.35±0.15
α-terpineol	0.87±0.15	0.21±0.07	0.17±0.04	0.14±0.04
**Sulphur compound**				
methyonol	0.13±0.04	0.16±0.04	0.30±0.11	0.22±0.05

The concentrations are expressed in mg/L; ±sd: standard deviation; nd: not detected

Table S2. The concentration of volatile compounds determined in the four wines obtained by the industrial-scale vinifications during the vintage 2018. The vinifications were carried out by separately inoculating in the same grape must with the mixed starter formulation (Mix_start) or by the sequential inoculation of three different commercial yeast preparations followed by the addition of a commercial malolactic starter at the end of the alcoholic fermentation (Comm).

Compounds	Mixstart_2018	Comm 1_2018	Comm 2_2018	Comm3_2018
**Esters**				
ethyl butanoate	1.86±0.36	1.54±0.36	1.38±0.7	1.42±0.25
isoamyl acetate	0.83±0.25	0.16±0.04	0.20±0.05	0.14±0.04
ethyl hexanoate	0.67±0.16	0.28±0.11	0.35±0.06	0.41±0.07
ethyl lactate	2.32±0.34	0.28±0.05	0.36±0.10	0.38±0.04
ethyl octanoate	nd	0.05±0.02	0.08±0.02	0.04±0.02
ethyl decanoate	nd	0.33±0.11	nd	nd
diethyl succinate	2.84±0.34	0.13±0.04	0.15±0.05	0.11±0.03
phenyl ethyl acetate	0.11±0.03			
diethyl malate	0.14±0.05			
mono ethyl succinate	7.93±1.75	0.72±0.17	0.81±0.26	0.54±0.11
**Alcohols**				
2 methyl-1-propanol	1.13±0.05	0.50±0.17	0.71±0.25	0.70±0.15
3-methyl-1-butanol	28.73±4.38	16.26±2.95	20.46±5.17	17.76±3.94
1-hexanol	0.60±0.16	nd	nd	0.15±0.04
methyonol	0.27±0.06	0.06±0.02	0.17±0.05	0.11±0.03
benzylalcohol	0.91±0.21	0.33±0.06	0.47±0.06	0.51±0.06
phenylethanol	34.40±5.62	17.97±3.10	22.42±3.20	18.87±4.18
**Acids**				
3 methyl butanoic acid	0.54±0.11	0.15±0.04	0.34±0.04	0.28±0.04
2 methyl hexanoic acid	nd	0.45±0.06	0.18±0.06	0.51±0.11
hexanoic acid	1.51±0.16	0.35±0.10	0.33±0.10	0.36±0.08
octanoic acid	1.25±0.27	0.26±0.04	0.28±0.07	0.21±0.05
decanoic acid	0.45±0.07	0.65±0.06	0.71±0.16	0.75±0.15
**Terpenes**				
linalolo	0.87±0.34	0.21±0.04	0.13±0.04	0.16±0.03
α-terpineol	0.44±0.10	0.12±0.03	0.18±0.04	0.27±0.06
**Sulphur compound**				
methyonol	0.27±0.05	0.06±0.02	0.17±0.04	0.11±0.03

The concentrations are expressed in mg/L; ±sd: standard deviation; nd: not detected
